# Performance evaluation of a series-connected step-up/down partial power converter for battery energy storage applications

**DOI:** 10.1038/s41598-026-35857-z

**Published:** 2026-01-16

**Authors:** Qian Liu, Long Jing, Wenzheng Xu, Ruidong Sun, Xuezhi Wu, Jianhua Lei, Yongbo Zhang

**Affiliations:** 1 National Active Distribution Network Technology Research Center, Beijing Jiaotong University, Beijing, 100044 China; 2 Shenzhen Poweroak Newener Company Ltd, Shenzhen, 516083 China

**Keywords:** Full power converter (FPC), Battery energy storage applications, Step-up/down partial power converter (SUDPPC), Nonactive power, Component stress factor (CSF), Energy science and technology, Engineering

## Abstract

The conventional full power converter (FPC) for battery energy storage applications is limited by bulky components and suboptimal efficiency. In response, a series-connected step-up/down partial power converter (SUDPPC) with high power density is proposed in this paper. It consists of an LLC resonant converter operating at a fixed switching frequency cascaded with a full-bridge converter capable of providing bipolar output. By connecting the SUDPPC in series with the load, the voltage stress on the series side and the current stress on the parallel side are markedly reduced. The four-quadrant function provides support for further optimization of the rated power level. Universal series interconnection schemes are elaborated, and design guidelines are formulated based on power distribution characteristics. Furthermore, the topology is evaluated in terms of nonactive power and component stress factor (CSF), and benchmarked against a four-switch buck/boost FPC and a phase-shifted full-bridge step-up partial power converter (SUPPC). Finally, a 1.1 kW prototype is developed to experimentally validate the theoretical analysis, demonstrating that only 14.3% of the total active power is processed under full-load conditions, with a peak efficiency of 98.15%.

## Introduction

In emerging battery energy storage applications, the bidirectional dc-dc converter serves as a critical interface between the battery and the dc bus, with its performance exerting a significant impact on overall system efficiency^1,2^. Depending on voltage levels, noise sensitivity, and current requirements, both isolated topologies using high-frequency transformers and non-isolated configurations^3,4^ may be adopted. However, conventional parallel schemes are based on full power processing, in which the voltage and current stresses of components are governed by steady-state power, resulting in elevated conduction losses^5^.

**Fig. 1:**
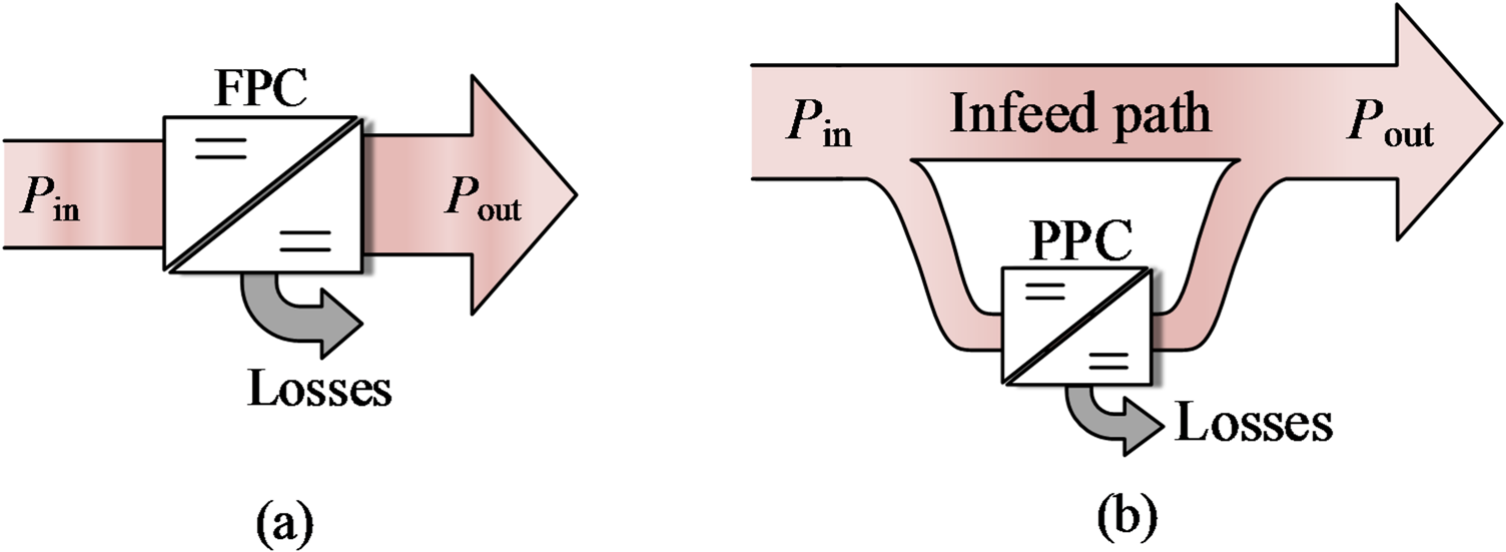
Schematic diagram of system power flow with (**a**) FPC and (**b**) PPC.

In contrast, partial power processing (PPP) has been recognized as a promising approach for achieving compact and efficient regulation^6^. Its primary goal is to reduce component ratings, physical dimensions, and power losses^7^. Rather than refining converter topologies, soft-switching techniques, or control strategies, PPP systems are characterized by a direct infeed path that transfers the majority of power^6,8,9^. As shown in Fig. 1, only a small fraction of the total power is processed by the series-connected partial power converter (PPC), while the remainder is delivered to the load without conversion loss^10–12^. Consequently, regulation is accomplished using low-rated components, leading to notable enhancements in power density and conversion efficiency^13–15^. This unique connectivity makes the PPC particularly suitable for applications such as traction powertrains, photovoltaic (PV) systems, battery energy storage, and data-center power delivery.

However, the effectiveness of converter series operation in fulfilling PPP has been questioned in previous studies. In certain implementations, the PPC reduces only the active power flow while neglecting the reactive component, which may lead to decreased overall system efficiency^16,17^. This inefficiency stems from the interaction between active and nonactive power, which jointly influence component losses. Moreover, even when PPP is successfully realized, optimal performance requires careful consideration of trade-offs among connectivity configurations, topology choices, and parameter design.

Most of the works carried out in PPC-based are focused on either step-up (SUPPC) or step-down (SDPPC) operation. If the series port (SP) voltage is designed as bipolar, i.e., the conversion system is managed by SUDPPC, the power processing level can be further reduced. In^18^, a SUDPPC intended for offshore wind farm applications is implemented using a dual active bridge converter combined with an unfolder circuit, which only inverts the SP voltage polarity without providing active regulation. Consequently, the transition near zero-voltage becomes challenging, though it remains essential in specific operational scenarios. In^10^, four-quadrant switches are configured on the secondary side of a phase-shifted full-bridge (PSFB) converter, where the negative output voltage is achieved by forcing the inductor current to flow back into the input source. However, this method is characterized by a complex dynamic model, making it relatively challenging to control and analyze. Similar concepts are also discussed in^19–22^. Another SUDPPC topology with mode switching achieved via additional switches is conducted in^23^, where control must be temporarily interrupted to avoid overshoot as the bias voltage approaches zero. It should be noted that none of the above studies systematically evaluated the effectiveness of PPP.

To present the relevant content, a SUDPPC topology consisting of an LLC resonant converter and a full-bridge (FB) converter is developed in this paper. The LLC operates in a high-efficiency resonant state and provides galvanic isolation, while the FB converter achieves system regulation by managing the SP voltage or current. The topology is evaluated in terms of nonactive power and the component stress factor (CSF), and a detailed comparative analysis is conducted with a unipolar SUPPC employing PSFB and a four-switch buck-boost (FSBB) full power converter (FPC). The results indicate that the SUDPPC yields the lowest total circulating energy and the highest component utilization. In addition, the universal connection schemes and corresponding selection guidelines for PPC are presented. Finally, the modeling and control design of the proposed SUDPPC are presented, followed by hardware verification and conclusions.

## Interface configurations and performance metrics for PPC

To support configuration selection tailored to specific requirements, universal interface schemes and associated power distribution characteristics are examined. Besides, key performance metrics for evaluating different topologies are introduced.

### Classification of interface configurations

**Fig. 2:**
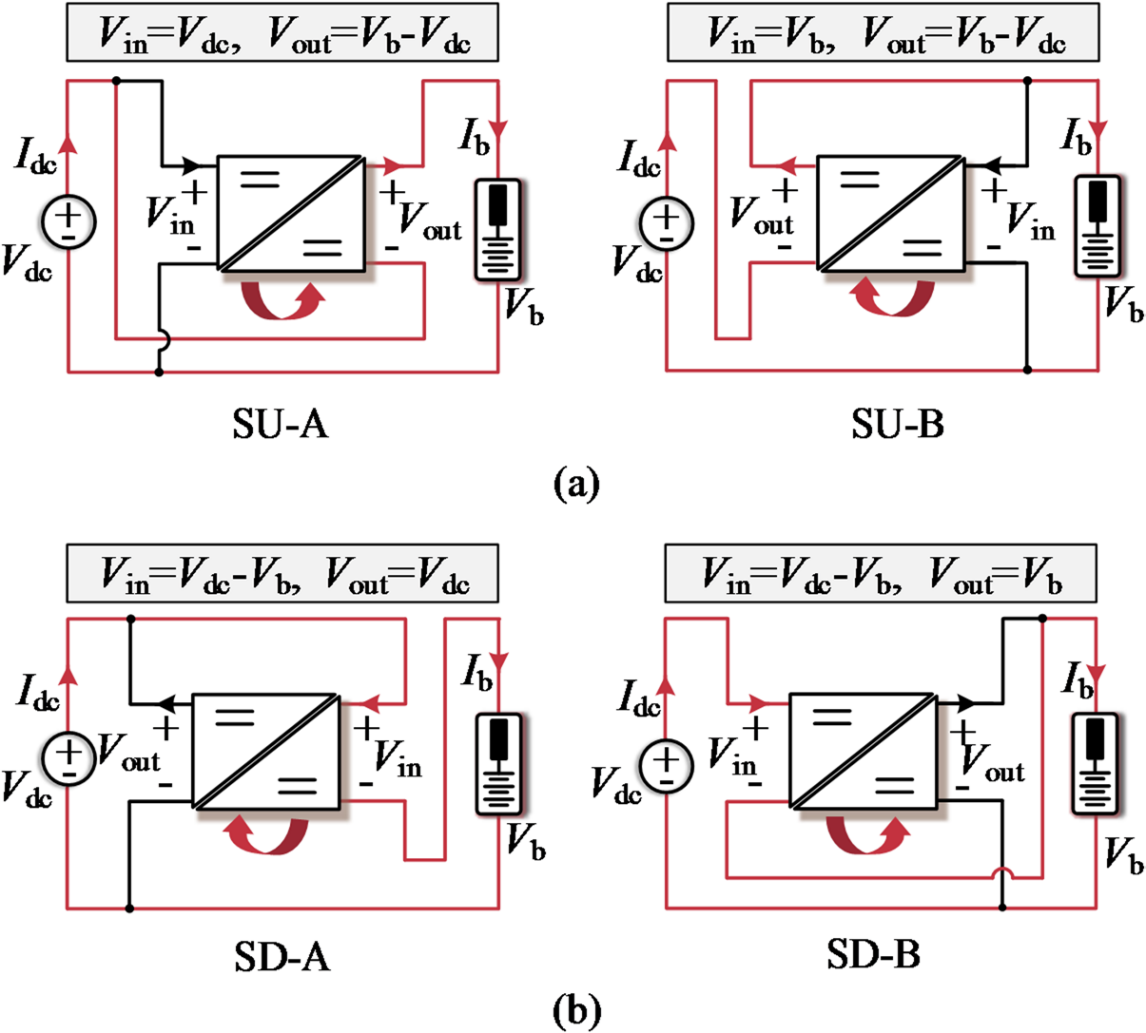
The step-up and step-down configurations of PPC are denoted by (**a**) SU-A/SU-B and (**b**) SD-A/SD-B, respectively.

The PPC functions as a power shunt, with one port connected in series between the battery and the dc bus, while the other port is connected in parallel to either side. As depicted in Fig. 2, PPCs are primarily categorized into step-up and step-down types, with the series path and corresponding power flow direction indicated by red lines and arrows. Without loss of generality, the battery pack is assumed to be in a charging state.

In Fig. 2, the PPC connected in parallel with the dc bus is categorized as type A, otherwise it is designated as type B. Here *V*_dc_ and *I*_dc_ represent the dc bus voltage and current, while *V*_b_ and *I*_b_ denote the battery voltage and current, respectively. It is obvious that the operating conditions of PPC are strongly influenced by the interface configuration. The SU-A configuration can be derived by changing the SP voltage polarity of the SD-A, which is accompanied by a reversal of the converter power flow direction. Therefore, when the SP voltage exhibits bipolar characteristics, SU-A and SD-A (as well as SU-B and SD-B) possess identical power handling capabilities, and bidirectional switching devices are required.

It should be emphasized that a shared reference ground is inherently formed between the input source and the load in a series-connected PPC. Nevertheless, utilizing an isolation transformer is strongly recommended to mitigate short-circuit risks. As mentioned in^10^, Rakoski Zientarski et al. demonstrated that only isolated converters with non-unity transformer turns ratio can fully realize PPP. In fact, minimizing the stress on the switching device is essential for ensuring proper PPP operation. Significant reduction in conduction losses can only be achieved by simultaneously avoiding rated voltage and current, a challenge effectively addressed by the series configuration of isolation topologies.

### Active power processing

Based on the analysis conducted in this study, the PPC characteristics are investigated using the SD-A as a representative case. The SP shares the same current as the battery, and its blocking voltage is denoted as Δ*V* (Δ*V* =|*V*_dc_-*V*_b_|). Therefore, the power relationship of the SD-A can be described as1

where *P*_out_ is the output active power, and *P*_d_ is the active power delivered by the direct infeed path. *P*_p_​ refers to the active power processed by the PPC, which may be unidirectional or bidirectional. *V*_2_ represents the voltage of SP.

The allocation mechanism for power transfer can be defined by Eq. (2).

2 $$\begin{gathered} {\mathrm{PPF=}}{P_{\mathrm{p}}}{\mathrm{/}}{P_{{\mathrm{out}}}}{\mathrm{=}}\Delta V{\mathrm{/}}{V_{\mathrm{b}}} \hfill \\ {\mathrm{PP}}{{\mathrm{F}}^{\mathrm{*}}}{\mathrm{=}}{P_{\mathrm{d}}}{\mathrm{/}}{P_{{\mathrm{out}}}}{\mathrm{=}}{V_{{\mathrm{dc}}}}{\mathrm{/}}{V_{\mathrm{b}}} \hfill \\ \end{gathered}$$

where the power processing factor (PPF) and PPF^*^ indicate the fractions of active power processed by the PPC and the direct infeed path, respectively. When PPF equals zero, energy is transmitted solely through the direct infeed path, achieving an ideal efficiency of 100%. As the PPF increases and gradually becomes greater than unity, the system rated or higher active power is processed by the converter, contradicting the principle of PPP.

Here, *V*_dc_ is normalized to unity, and Δ*V* is conceived as a dimensionless parameter that varies with the battery voltage. Following similar calculations, the PPF and PPF^*^ considering the bipolar SP voltage of the PPCs are summarized in Table 1. It is clear that a smaller Δ*V* facilitates a lower converter power level. When the directly fed power exceeds the system rating (i.e., PPF^*^ > 1), the surplus is pushed back to the input through the converter, thereby increasing the processed power. For instance, with a positive SP voltage, the SU-B exhibits a higher PPF compared to SU-A. Hence, PPCs should be selected according to specific criteria to ensure operation within an optimal voltage range.

**Table 1:** PPF and PPF* of PPCs with bipolar SP voltage.

SP voltage polarity	PPF		PPF*	
	Positive	Negative	Positive	Negative
SU-A	Δ*V*/(1 + Δ*V*)	Δ*V*/(1-Δ*V*)	1/(1 + Δ*V*)	1/(1 + Δ*V*)
SU-B	Δ*V*	Δ*V*	1 + Δ*V*	1-Δ*V*
SD-A	Δ*V*/(1-Δ*V*)	Δ*V*/(1 + Δ*V*)	1/(1-Δ*V*)	1/(1 + Δ*V*)
SD-B	Δ*V*	Δ*V*	1-Δ*V*	1 + Δ*V*

**Fig. 3:**
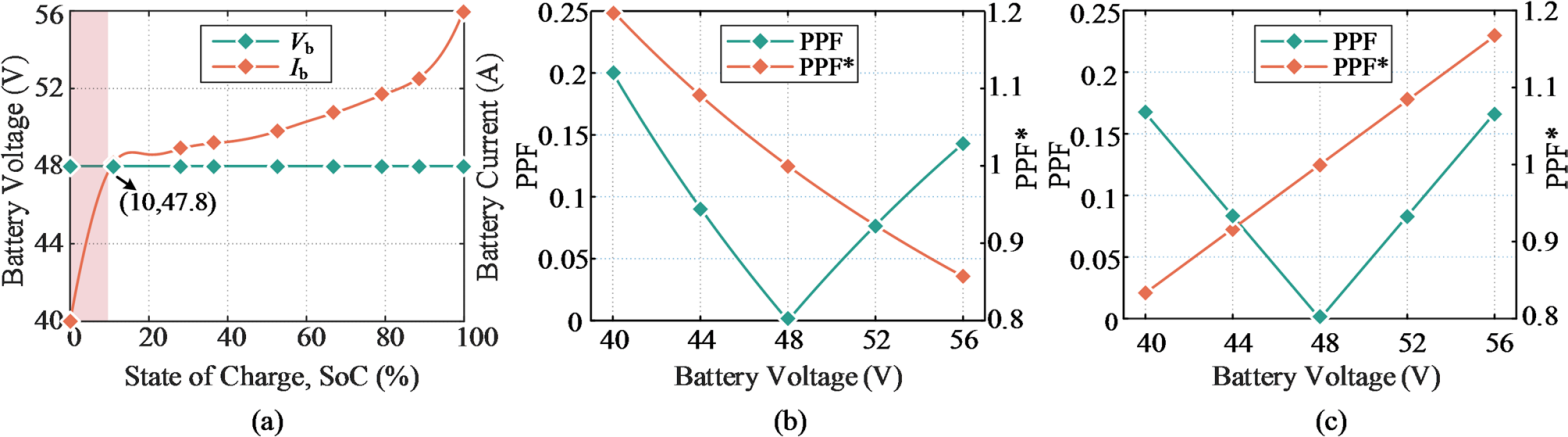
Power distribution characteristics of SUDPPC guided by the adopted lithium-ion battery. (**a**) Typical CC charging curve depicted by interpolation method. (**b**) Performance of SU-A/SD-A. (**c**) Performance of SU-B/SD-B.

This study is conducted using a 48 V dc bus within a low-voltage power system, commonly utilized in residential energy storage systems, communication infrastructure, and data centers. To achieve optimal voltage matching, a 50 Ah lithium-ion battery from LG Chem is employed, as shown in Fig. 3(a), with a voltage range of 40–56 V. Constant current (CC) control is implemented to ensure stability and predictability in energy transfer between the battery and the dc bus. To mitigate performance degradation, the battery is operated above a 10% state of charge (SOC), as generally recommended. The power distribution characteristics of SU-A/SD-A and SU-B/SD-B under bipolar SP voltage conditions are illustrated in Figs. 3(b) and (c). Only 14.3% of the total active power is handled by SU-A/SD-A at full load, compared to 16.7% by SU-B/SD-B., indicating that SU-A/SD-A offers a more size and stress efficient component design. Additionally, in the SU-B/SD-B configuration, the battery current is split between the input and output branches of the PPC, complicating the implementation of CC control.

### Performance evaluation metrics

The switching actions of semiconductor devices induce internal energy circulation without delivering power to the load, introducing the concept of nonactive power^10^. In PPC, achieving high efficiency presupposes that the nonactive power is also partially processed, since it represents the circulating energy that affects the component size and losses. As discussed in^16^, PPC implemented with a buck-boost converter exhibits nearly identical circuit pattern and efficiency performance as a conventional boost converter, even though its PPF is below unity. Moreover, a similar behavior is observed in a series-connected flyback converter^23^, where the coupled inductors transfer more nonactive power than the isolation transformer. These systems are fundamentally constrained from achieving PPP due to excessive nonactive power circulation, substantially degrading converter efficiency.

**Table 2:** Values of V^*^ and I^*^ for components.

PPC configuration	V*	I*
Capacitors	Average voltage	RMS current
Inductors	Average ac voltage	RMS current
Transformers	Average ac voltage	RMS current
Diodes	Blocking voltage	Average current
MOSFETs	Blocking voltage	RMS current

For inductors and capacitors, the nonactive powers *N*_*L*_ and *N*_*C*_ ​can be calculated based on the energy absorbed and released during the switching period *T*_*s*_​^17^.

3 $$\begin{gathered} {N_L}=\frac{{\int_{0}^{{\alpha {T_s}}} {\left| {{v_L}(t){i_L}(t)} \right|dt} +\int_{{\alpha {T_s}}}^{{{T_s}}} {\left| {{v_L}(t){i_L}(t)} \right|dt} }}{{{T_s}}} \hfill \\ {N_C}=\frac{{\int_{0}^{{\alpha {T_s}}} {\left| {{v_C}(t){i_C}(t)} \right|dt} +\int_{{\alpha {T_s}}}^{{{T_s}}} {\left| {{v_C}(t){i_C}(t)} \right|dt} }}{{{T_s}}} \hfill \\ \end{gathered}$$

where $\alpha {T_s}$ is the moment of transition between energy absorption and release. Note that for the passive component responsible for filtering, Eq. (3) can be simplified to Eq. (4) using the small ripple approximation.

4 $$\begin{gathered} {N_L}=\frac{{2L \cdot {{\bar {i}}_L} \cdot \Delta {i_L}}}{{{T_s}}} \hfill \\ {N_C}=\frac{{2C \cdot {{\bar {v}}_c} \cdot \Delta {v_c}}}{{{T_s}}} \hfill \\ \end{gathered}$$

where ${\bar {i}_L}$​ and $\Delta {i_L}$ represent the average current and current ripple of the inductor, and ${\bar {v}_c}$​ and $\Delta {v_c}$ represent the average voltage and voltage ripple of the capacitor. The total nonactive power managed by the PPC is expressed as the sum of the aforementioned components. Full compliance with the PPP principle is achieved only when both active and nonactive power in the conversion system are partially processed under identical operating conditions.

Besides, as a key complementary metric for evaluating converter performance^24–27^, the CSF provides a dimensionless result by calculating the apparent power of each component.

5 $${\mathrm{CSF}}=\frac{{{I^*}{V^*}}}{{{P_{{\mathrm{out}}}}}}$$

where V* and I* are determined depending on the component properties, as shown in Table 2.

Based on the values presented in Table 2, the voltage and current stresses of the components are related by the CSF, which indirectly serves as an auxiliary metric for comparing conduction losses across different topologies. Therefore, weight factors can be assigned according to the component on-resistance to adapt to different scenarios, and they are set to 1 in this paper.

**Fig. 4:**
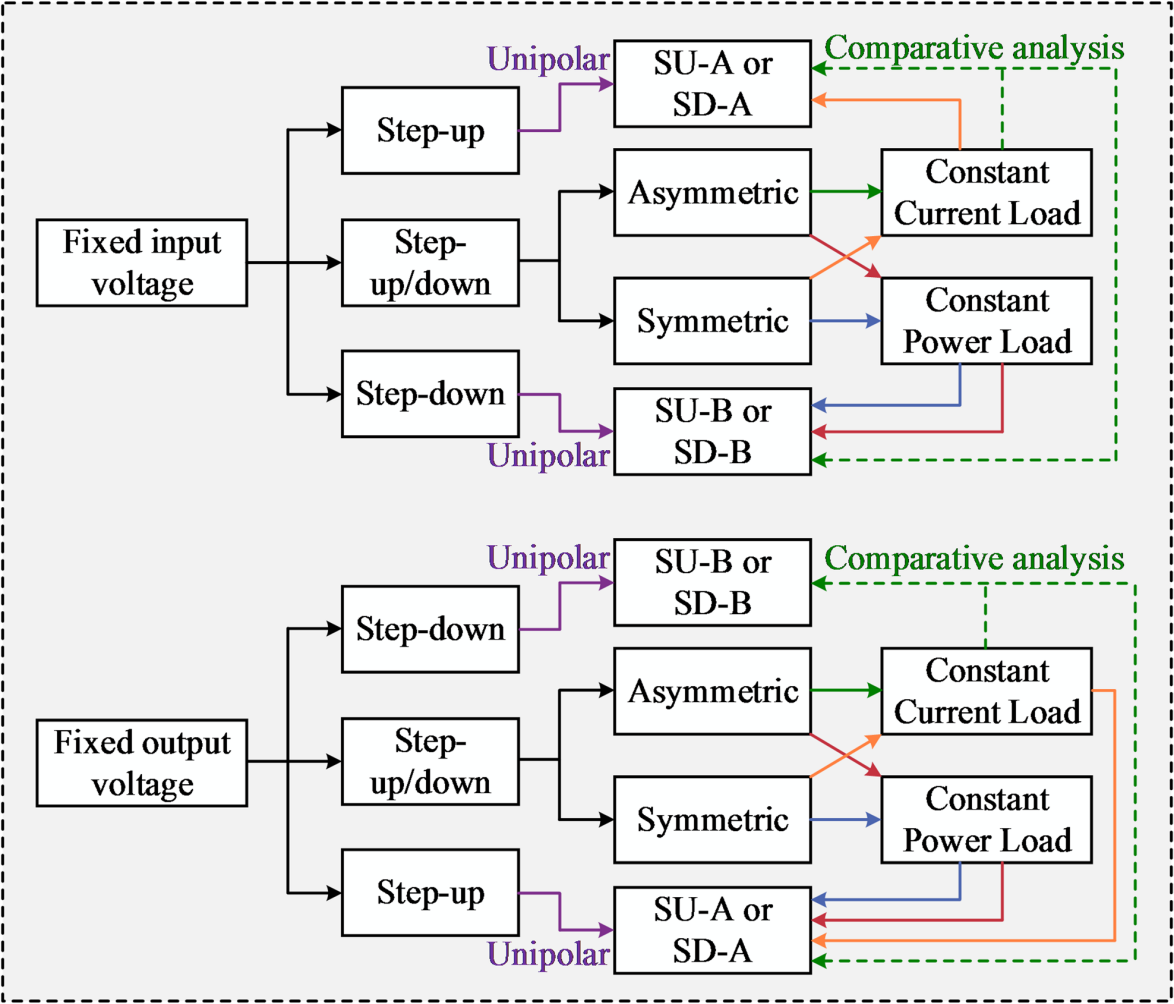
Guidelines for PPC configuration selection.

### Configuration selection guidelines

Indeed, reducing the maximum power rating of the PPC should be prioritized in configuration selection, as it dictates component specifications^28^. In most practical scenarios, system input or output parameters are typically fixed, such as a stable dc bus voltage in battery charging systems^29^ or a regulated output voltage in onboard electronics. Additionally, the load may exhibit CC^30^ or constant-power^31,32^ characteristics. Based on the analysis presented in Table 1, selection guidelines for PPCs are systematically derived and illustrated in Fig. 4, with key conclusions summarized below.

i. Unipolar PPC: For systems operating with a positive SP voltage, SU-A is preferred for step-up applications, whereas SD-B is more suitable for step-down conversion. In contrast, under a negative SP voltage, SD-A and SU-B are optimally employed for step-up and step-down operations, respectively. A review of previous studies reveals that SU-B has been utilized in certain unidirectional step-up systems with positive SP voltage^33^, while a similar misconception is observed in step-down converters implemented with SD-A^31,34^. Although these PPCs enable substantial reductions in processed power, they remain limited by issues related to power backflow.
ii. Bipolar PPC with constant power loads: When the input voltage is fixed, configurations based on the SU-B/SD-B scheme are more suitable, as they yield the lowest peak PPF across the output voltage range [see Fig. 3(c)]. Conversely, SU-A/SD-A enables reduced converter ratings when the output voltage is held constant. In summary, the port with variable voltage should be preferentially selected for parallel connection.
iii. Bipolar PPC with CC loads: When the voltage difference is symmetrically distributed between the positive and negative directions, SU-A/SD-A performs superiorly in handling input/output voltage variations. In cases of asymmetric voltage distribution, a comparative analysis supported by detailed calculations is required.

It is worth noting that the guidelines in Fig. 4 are applicable across different power and voltage levels, and the optimal PPC configuration can therefore be selected according to the operating conditions. For instance, in a dc-dc converter with a given rated power and fixed input voltage, SU-B and SD-B are generally preferred when the input and output voltage ranges overlap, independent of the specific voltage and power levels.

Despite its broad application prospects, the PPC topology exhibits pronounced audio susceptibility to input variations due to its direct feed path, which allows disturbances to be directly coupled to the output. As a result, advanced control schemes with strong disturbance-rejection capability are desirable for PPCs. Moreover, the series operation of PPC results in a narrow voltage regulation range, making it unsuitable for high-ratio applications. On the other hand, implementing the SUDPPC with a bipolar port requires additional power switches, which introduces a trade-off between operating efficiency and hardware cost.

## Analysis of the proposed SUDPPC

In view of the preceding analysis, the SD-A configuration is adopted as the optimal power level implementation for the SUDPPC, with its operating principles, parameter tuning, and control design discussed in this section.

### Operating principle

**Fig. 5:**
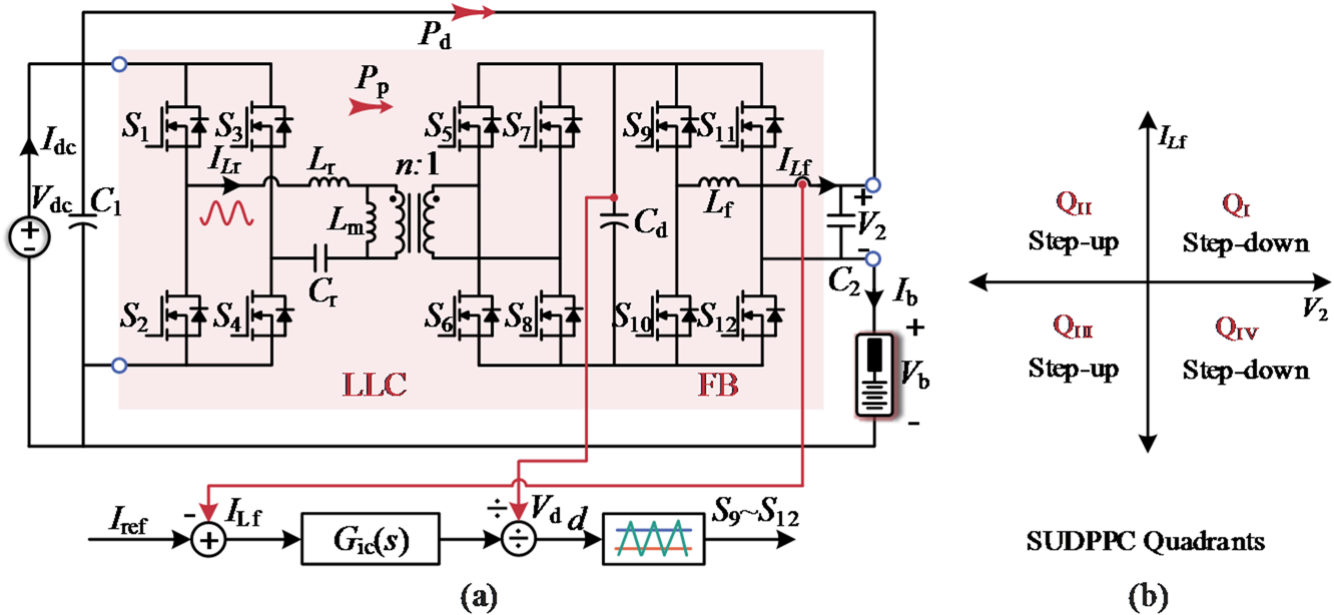
Schematic diagram of the (**a**) topology of the studied SUDPPC case and its (**b**) four-quadrant operation.

Figure 5 illustrates the proposed SUDPPC topology and its four-quadrant operation, where the front-stage is equipped with an LLC converter operating in open-loop resonant point to provide current isolation and soft-switching capabilities. The output voltage (i.e., SP voltage) *V*_2_​​ of the subsequent FB converter is determined by the equivalent duty cycle *d*, i.e., *V*_2_ = *V*_dc_⋅*d*/*n*. The value of *d* ranges from − 1 to 1, and *n* is the turns ratio of the transformer. Current control is achieved using a current-loop scheme with a high-bandwidth proportional-integral (PI) controller, *G*_ic_(*s*).

From the series relationship in Fig. 5, the input to output voltage gain *G* can be expressed as:

6 $$G=\frac{{{V_{\mathrm{b}}}}}{{{V_{{\mathrm{dc}}}}}}=\frac{{n - d}}{n}$$

**Fig. 6:**
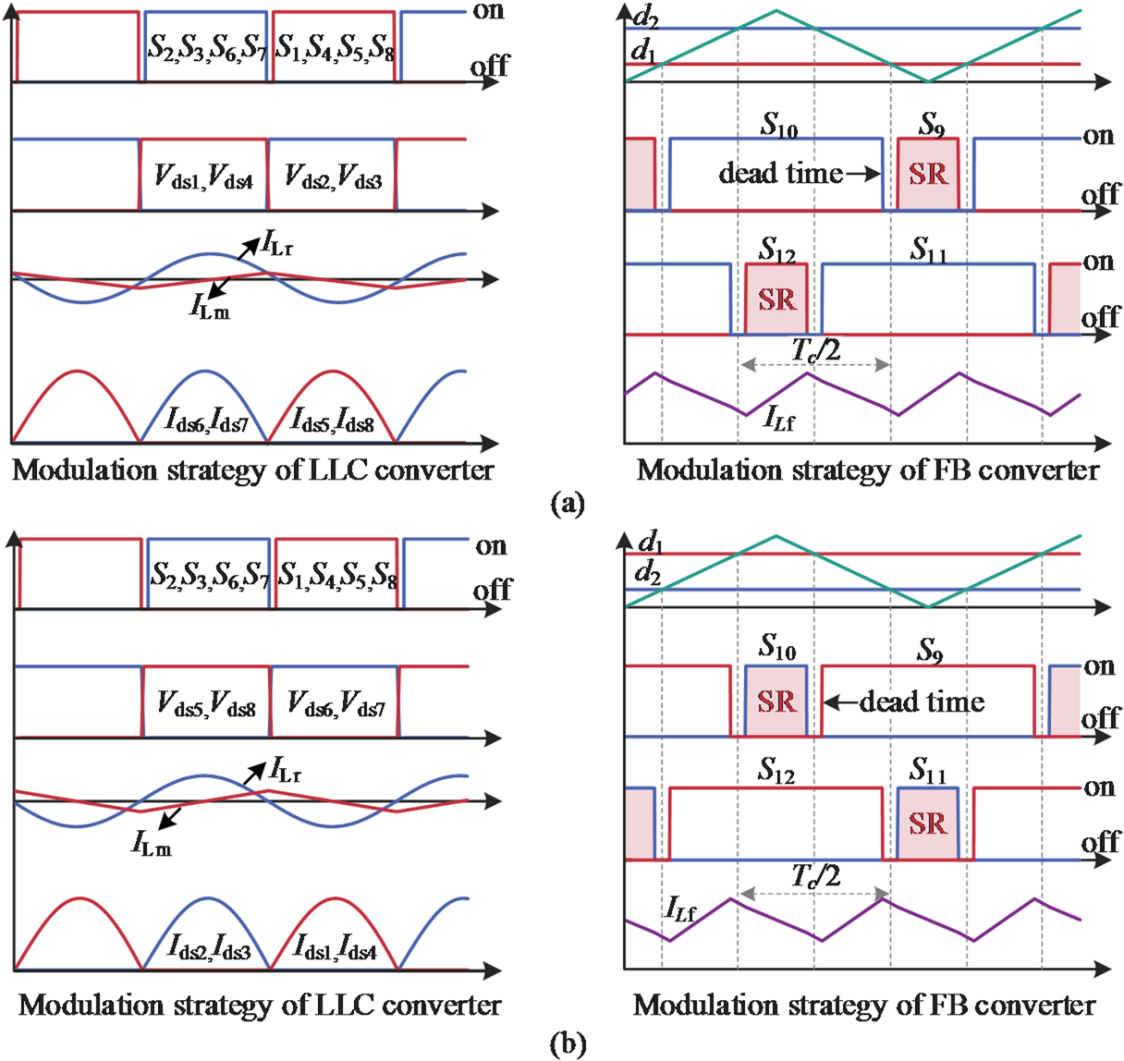
SUDPPC modulation scheme. (**a**) Step-up mode, with *V*_b_=52.8 V, *d*=-0.5, PPF = 0.09, and PPF∗=0.91. (**b**) Step-down mode, *V*_b_=43.2 V, *d* = 0.5, PPF = 0.11, and PPF∗=1.11.

Taking battery charging as an example, the key waveforms of the SUDPPC operating in step-up/down mode are shown in Fig. 6, where the red shaded area indicates the switches performing synchronous rectification (SR) in the FB converter. *I*_Lf​_, *I*_Lr_​, and *I*_Lm_ represent the currents flowing through the filter inductor *L*_f_​, the resonant inductor *L*_r_​, and the magnetizing inductor *L*_m_​, respectively. *V*_ds​_ and *I*_ds_ correspond to the voltage and current of power switch.

As indicated by the rectified current, the SUDPPC transfers power from the parallel side to the series side in step-up mode, whereas the power flow reverses in step-down mode. The sinusoidal resonant current waveform confirms that the LLC converter operates precisely at the resonant point. The FB converter employs unipolar frequency doubling modulation to reduce the effective period of the inductor current, thereby suppressing high frequency ripple. Specifically, MOSFETs *S*_9_ and *S*_11_ use different modulation waves to generate duty cycles *d*_1_ and *d*_2_, respectively, with the FB converter’s equivalent duty cycle given by *d = d*_1_-*d*_2_. The gate signals for the same leg are maintained in a logically complementary state, with an appropriate dead time inserted. Therefore, this topology can easily achieve zero-voltage stable output and four-quadrant operation.

### Parameter tuning

The transformer turns ratio *n* has a significant impact on circulating nonactive power and component utilization, and its maximum permissible value *n*_max_ is constrained by the duty ratio.

7 $${n_{\hbox{max} }}=\frac{{{V_{{\mathrm{dc}}}}\left| {{d_{\hbox{max} }}} \right|}}{{\left| {{V_{{\mathrm{b}}\hbox{min} /\hbox{max} }} - {V_{{\mathrm{dc}}}}} \right|}}$$

**Fig. 7:**
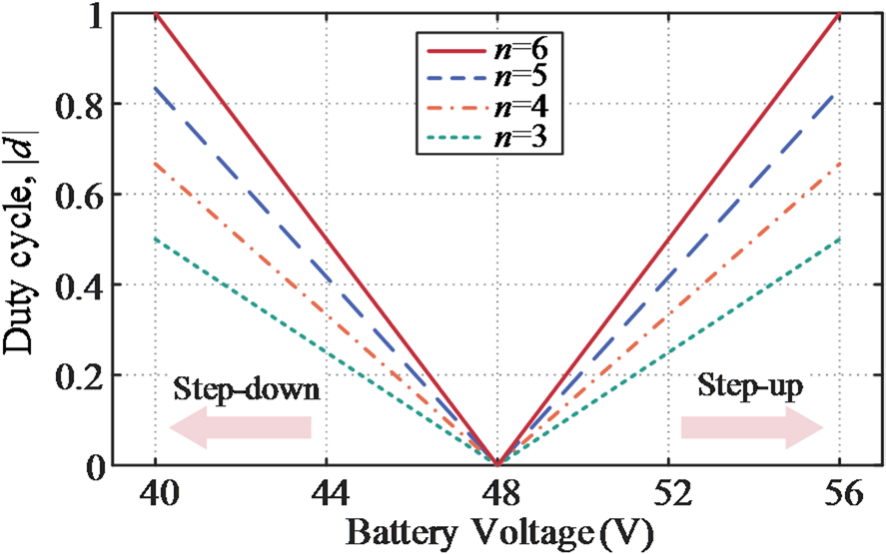
Duty cycle behavior for different values of *n*.

The duty cycle behavior for different values of *n* is reported in Fig. 7, where *n* = 5 is identified as an appropriate choice, providing a 16.7% control margin to account for non-idealities.

To satisfy the soft-switching in the LLC converter, *I*_Lm_​ must fully discharge the output capacitors *C*_oss_​ of the primary switches within the dead time *t*_*d​*_.

8 $${I_{{\mathrm{Lm}}}}=\frac{{{V_{{\mathrm{dc}}}}}}{{4{L_{\mathrm{m}}}{f_s}}} \geqslant \frac{{2{V_{{\mathrm{dc}}}}{C_{{\mathrm{oss}}}}}}{{{t_d}}}$$

Here, *f*_*s*_​ denotes the LLC converter switching frequency, which is set to 100 kHz in this paper, and *t*_*d*_ ​ is obtained from the double-pulse test and equals 200 ns. Accordingly, the constraints for *L*_m_​ is derived.

Further, the resonant capacitor *C*_r_ and *L*_r_ can be determined by Eq. (9).

9 $$Q=\frac{{{\pi ^2}{P_{\hbox{max} }}\sqrt {{L_{\mathrm{r}}}/{C_{\mathrm{r}}}} }}{{8V_{{{\mathrm{dc}}}}^{2}}},{\text{ }}{f_s}=\frac{1}{{2\pi \sqrt {{L_{\mathrm{r}}}{C_{\mathrm{r}}}} }}$$

where *P*_max_ is the maximum power processed by SUDPPC, and the quality factor *Q* is taken as 0.57.

The peak values ​​of *I*_*L*r_ and the resonant capacitor voltage *V*_*C*r_ can be further obtained^35^:

10 $$\begin{gathered} {I_{Lr\hbox{max} }}=\frac{{{P_{\hbox{max} }}}}{{4n{V_{\mathrm{d}}}}}\sqrt {4{\pi ^2}+\frac{{{n^4}{V_{\mathrm{d}}}^{4}}}{{L_{{\mathrm{m}}}^{2}f_{s}^{2}P_{{\hbox{max} }}^{2}}}} \hfill \\ {V_{Cr\hbox{max} }}=\frac{{{P_{\hbox{max} }}}}{{4n{V_{\mathrm{d}}}{f_s}{C_{\mathrm{r}}}}} \hfill \\ \end{gathered}$$

where *V*_d_ is the output voltage of the LLC converter.

For FB converter, the current ripple Δ*I*_Lf_ of the *L*_f_ is constrained to within 10% to mitigate cumulative degradation effects on the battery pack, as expressed by the following requirements.

11 $${L_{\mathrm{f}}} \geqslant \frac{{{V_{\mathrm{d}}} - \left| {{V_{{\mathrm{dc}}}} - {V_{\mathrm{b}}}} \right|}}{{2\Delta {I_{L{\mathrm{f}}}}{f_c}}} \cdot \frac{{\left| {{V_{{\mathrm{dc}}}} - {V_{\mathrm{b}}}} \right|}}{{{V_{\mathrm{d}}}}}$$

The switching frequency *f*_*c*_ is chosen as 50 kHz to minimize switching losses. With regard to the filter capacitors *C*_1_, *C*_d_, and *C*_2_, their values are determined by the 0.5% voltage ripple:

12 $${C_1}=\frac{{{P_{\hbox{max} }}}}{{2V_{{{\mathrm{dc}}}}^{{}}{f_s}\Delta {V_{{\mathrm{dc}}}}}},{\text{ }}{C_{\mathrm{d}}}=\frac{{{P_{\hbox{max} }}}}{{2{V_{\mathrm{d}}}{f_s}\Delta {V_{\mathrm{d}}}}},{\text{ }}{C_2}=\frac{{\Delta {I_{L{\mathrm{f}}}}}}{{16{f_c}\Delta {V_2}}}$$

Since the SP sustains a small bias voltage, *C*_2_ is designed with a increased capacitance to ensure compliance with voltage ripple constraints. However, due to the high power density of capacitors, this requirement does not adversely affect the system volume. Furthermore, the bipolar characteristic permits the adoption of compact and highly reliable film capacitors for *C*_2_.

For the selection of power devices, the blocking voltage of MOSFETs *S*_1_-*S*_4_​ is *V*_dc_, whereas the blocking voltage of *S*_5_​-*S*_12_​ is *V*_dc_​/*n*. In addition, the current stress of *S*_1_-*S*_4_​​ is the peak value of *I*_*L*r_, the current stress of *S*_9_​-*S*_12_​​ equals the battery current *I*_b_, and the current stress of *S*_5_​-*S*_8_​ can be expressed by:

13 $${I_{{\mathrm{dsmax(5-8)}}}}=\frac{{\pi {P_{\hbox{max} }}}}{{2{V_{\mathrm{d}}}}}$$

The root-mean-square (RMS) value of the current required for calculating the losses of the power devices can then be obtained, as given in Eq. (14).

14 $$\begin{gathered} {I_{{\mathrm{dsrms(1-4)}}}}=\frac{{{I_{Lr\hbox{max} }}}}{{\sqrt 2 }}{\text{, }}{I_{{\mathrm{dsrms(9,12)}}}}={I_{\mathrm{b}}}\sqrt {\frac{{{V_{\mathrm{d}}}+{V_2}}}{{2{V_{\mathrm{d}}}}}} \hfill \\ {I_{{\mathrm{dsrms(5-8)}}}}=\frac{{\pi {P_{\hbox{max} }}}}{{4{V_{\mathrm{d}}}}},{\text{ }}{I_{{\mathrm{dsrms(10,11)}}}}={I_{\mathrm{b}}}\sqrt {\frac{{{V_{\mathrm{d}}} - {V_2}}}{{2{V_{\mathrm{d}}}}}} \hfill \\ \end{gathered}$$

**Fig. 8:**
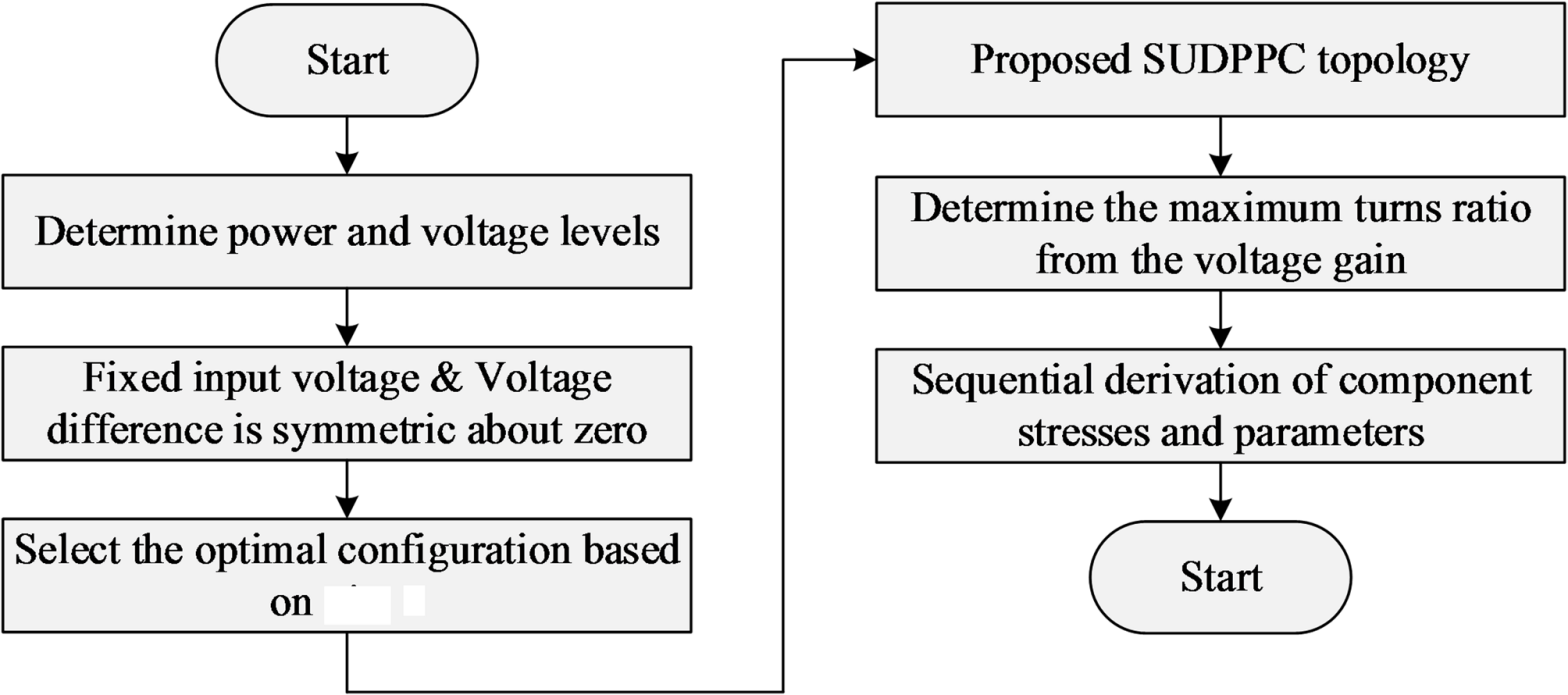
Flowchart of the proposed topology design.

In the PPC structure, the voltage stress on the series side and the current stress on the parallel side can be significantly reduced. By increasing the value of *n*, the blocking voltage of the series side semiconductor can be further reduced. Considering the above calculation process, the detailed specifications of the proposed SUDPPC are given in Table 3. Regarding the extension of the proposed SUDPPC to different power and voltage levels, a clear design flowchart is presented in Fig. 8. Most importantly, the transformer turns ratio *n* should be selected as the maximum value that satisfies the entire required voltage-gain range while maintaining sufficient control margin.

**Table 3:** Detailed specifications of the proposed SUDPPC.

Parameter	Part
Dc bus (*V*_dc_) and battery (*V*_b_) voltage	48 and (40–56) V
PPC maximum power (*P*_max_)	160 W
Filter capacitor (*C*_1_, *C*_d_, and *C*_2_)	0.07, 1.7, and 0.1 mF
Resonant inductor (*L*_r_) and capacitor (*C*_r_)	10.85 µH and 240 nF
Magnetizing inductor (*L*_m_)	200 µH
Filter inductor (*L*_f_)	12.5 µH
LLC converter switching frequency (*f*_s_)	100 kHz
FB converter switching frequency (*f*_c_)	50 kHz
MOSFETs (*S*_1_-*S*_4_, maximum stresses: 48 V/5.4 A)	BSC0803LSATMA1
MOSFETs (*S*_5_-*S*_8_, maximum stresses: 9.6 V/26 A)	IRLR6225PBF
MOSFETs (*S*_9_-*S*_12_, maximum stresses: 9.6 V/20 A)	IRLR6225PBF

### Control design

**Fig. 9:**
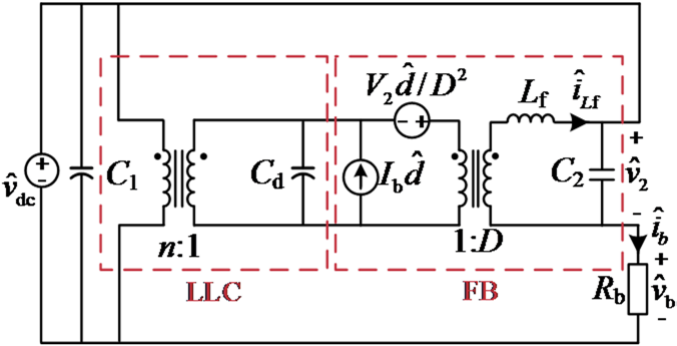
Small-signal model of SUDPPC under duty cycle perturbation.

Since the FB converter operates with controlled current, its dynamic model can be derived using the state-space averaging method. As for the front-stage LLC converter, it can be considered an ideal dc transformer. Therefore, the small-signal model of SUDPPC under duty cycle perturbation can be plotted in Fig. 9, where *D*, *V*_2​_ and *I*_b_ are the associated static values.

By analyzing the duty cycle disturbance $\hat {d}$ and the voltage and current equations at each node, the transfer function *G*_id_(*s*) of the controlled plant can be derived.

15 $${G_{{\mathrm{id}}}}(s)=\frac{{{{\hat {i}}_{L{\mathrm{f}}}}}}{{\hat {d}}}=\frac{{{V_2}({R_{\mathrm{b}}}{C_{\mathrm{2}}}s+1)}}{{D({L_{\mathrm{f}}}s+{R_b}({L_{\mathrm{f}}}{C_{\mathrm{2}}}{s^2}+1))}}$$

**Fig. 10:**
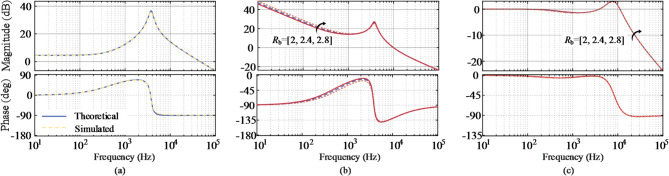
(**a**) Validation of the transfer function *G*_id_(*s*) from the control input to the inductor current. (**b**) Bode plot of open-loop transfer function. (**c**) Bode plot of closed-loop transfer function.

**Table 4:** Detailed specifications of the FSBB converter.

Parameter	Part
Dc bus (*V*_dc_) and battery (*V*_b_) voltage	48 and (40–56) V
PPC maximum power (*P*_max_)	320 W
Filter capacitor (*C*_1_ and *C*_2_)	334 and 200 µF
Filter inductor (*L*_f_)	66 µH
Switching frequency (*f*_s_)	100 kHz
MOSFETs *S*_1_-*S*_2_ (maximum stresses: 48 V/20 A)	IPD50N10S3L16
MOSFETs *S*_3_-*S*_4_ (maximum stresses: 56 V/23.3 A)	IPD50N10S3L16

**Table 5:** Detailed specifications of the PSFB converter.

Parameter	Part
Dc bus (*V*_dc_) and battery (*V*_b_) voltage	40 and (40–56) V
PPC maximum power (*P*_max_)	320 W
Filter capacitor (*C*_1_ and *C*_2_)	400 and 50 µF
Filter inductor (*L*_f_)	25 µH
Switching frequency (*f*_s_)	100 kHz
MOSFETs *S*_1_-*S*_4_ (maximum stresses: 40 V/10 A)	IAUZ20N08S5L300
MOSFETs *S*_5_-*S*_8_ (maximum stresses: 20 V/20 A)	BSZ097N04LSG

where *R*_b_​ represents the battery equivalent impedance, modeled as a resistor for simplicity. In particular, since the driving logic remains unchanged, the small-signal model of the SUDPPC is consistent in both step-up and step-down modes, simplifying the design of a unified controller.

An ac sweep analysis was conducted under the specified charging condition (*V*_b_=56 V, *I*_b_=20 A) using SIMPLIS software, with the results illustrated in Fig. 10(a). The simulated frequency response exhibits strong correlation with the theoretical prediction derived from (15), thereby validating the analytical model.

Based on a loop bandwidth of 10 kHz and a phase margin of 50 degrees, the PI regulator parameters are determined as *k*_p_=0.07 and *k*_i_=3700, ensuring excellent dynamic response and stability. The open-loop gain *L*(*s*) and closed-loop transfer function *T*(*s*) are then derived as follows:

16 $$L(s)={G_{{\mathrm{ic}}}}(s){G_{{\mathrm{id}}}}(s),{\text{ }}T(s)=\frac{{{G_{{\mathrm{ic}}}}(s){G_{{\mathrm{id}}}}(s)}}{{1+{G_{{\mathrm{ic}}}}(s){G_{{\mathrm{id}}}}(s)}}$$

Bode plots of *L*(*s*) and *T*(*s*) for different *R*_b_ are shown in Fig. 10(b) and (c), demonstrating that the high-bandwidth PI control offers strong robustness and stability against load variations.

## Performance evaluation and comparative analysis of different topologies

To comprehensively assess the advantages of the SUDPPC topology, a comparative analysis is conducted against conventional FPC and unipolar SUPPC topologies based on performance metrics and operational efficiency. The evaluation results are obtained from Eqs. (3) and (5), calculated using SIMPLIS, and SR is implemented with active power devices. A purely resistive load *R*_b_ is employed on the battery side to ensure a constant charging rate of 0.4 C. For consistency and validity, all comparison plants are designed with identical bias voltage ranges, ripple constraints, and load conditions, as detailed in Tables 4 and 5.

### Evaluation of the FSBB-based FPC

**Fig. 11:**
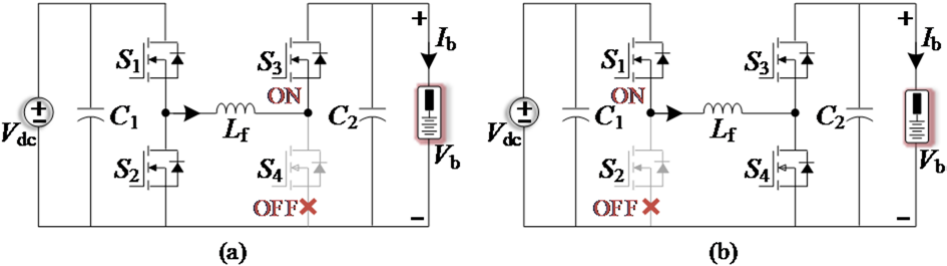
Schematic diagram of the FSBB-based FPC operating in (**a**) buck mode and (**b**) boost mode.

**Fig. 12:**
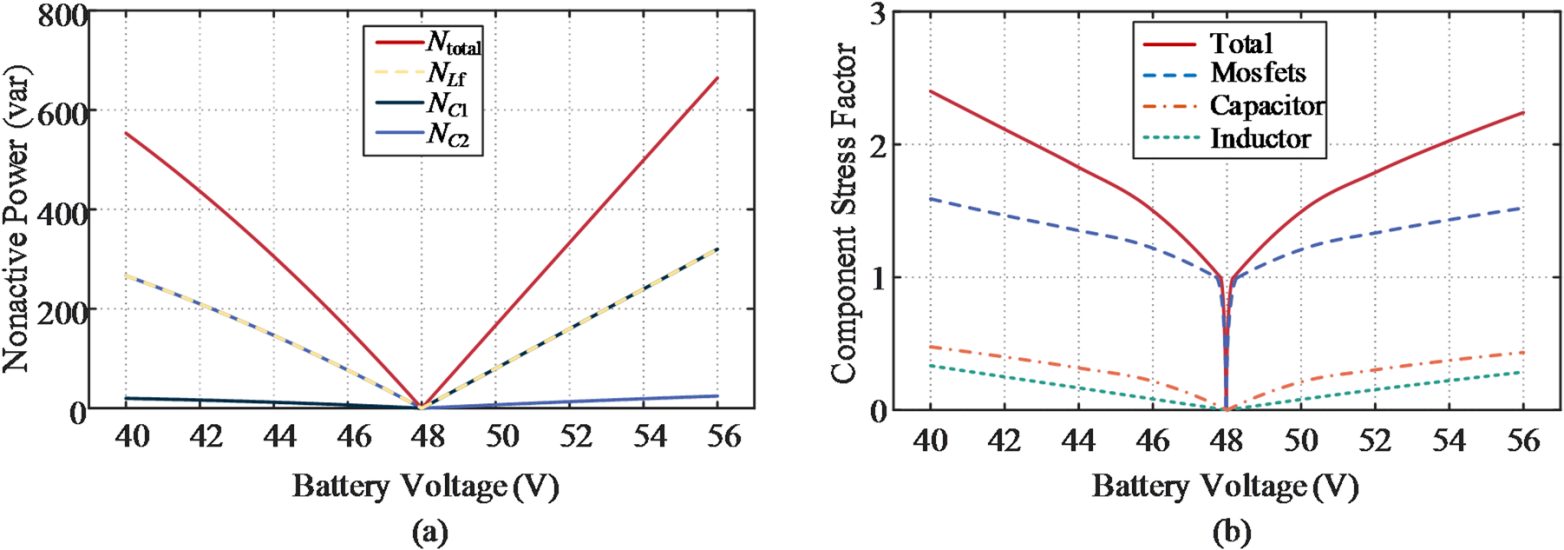
Detailed distribution of performance metrics for the FSBB-based FPC. (**a**) Nonactive power. (**b**) CSF.

The FSBB converter, with its non-isolation and step-up/down characteristics, serves as a benchmark for verifying the effectiveness of SUDPPC^31^. Its operating modes are divided into basic buck and boost circuits,

as shown in Fig. 11. Based on the small-ripple approximation in Eq. (4) and the operating principle of the FSBB converter, the expressions for nonactive power and CSF in both buck and boost modes are derived in Table 6. The curves obtained from this analytical model show strong consistency with the simulation results presented in Fig. 12.

**Table 6:** Simplified solution of performance evaluation metrics.

Parameter	Metric	C_1_	C_2_	L_f_	MOSFET
Buck mode:$D={V_{\mathrm{b}}}/{V_{{\mathrm{dc}}}}$	Nonactive power	$2P\left( {1 - D} \right)$	$\frac{{D{V_{\mathrm{b}}}({V_{{\mathrm{dc}}}} - {V_{\mathrm{b}}})}}{{4{L_f}{f_s}}}$	$2P\left( {1 - D} \right)$	None
	CSF	$\sqrt {\frac{{1 - D}}{D}}$	$\frac{{({V_{{\mathrm{dc}}}} - {V_{\mathrm{b}}})D}}{{\sqrt {12} {L_{\mathrm{f}}}{I_{\mathrm{b}}}{f_s}}}$	$2\left( {1 - D} \right)$	$\frac{{\sqrt D +\sqrt {1 - D} }}{D}$
Boost mode:$D=({V_{\mathrm{b}}} - {V_{{\mathrm{dc}}}})/{V_{\mathrm{b}}}$	Nonactive power	$\frac{{V_{{{\mathrm{dc}}}}^{{\mathrm{2}}}D}}{{4{L_{\mathrm{f}}}{f_s}}}$	$2PD$	$2PD$	None
	CSF	$\frac{{{V_{{\mathrm{dc}}}}D(1 - D)}}{{\sqrt {12} {L_{\mathrm{f}}}{I_{\mathrm{b}}}{f_s}}}$	$\sqrt {\frac{D}{{1 - D}}}$	$2D$	$\frac{{\sqrt D +\sqrt {1 - D} }}{D}$

Analysis indicates that, in buck mode, the filter inductor *L*_f_ and input capacitor *C*_1_​ contribute a significant and nearly equal amount of nonactive power. This arises from the inherent discontinuities in the voltage across *L*_f​_​ and the current passing through *C*_1_​, which generate higher harmonic components. By contrast, the output capacitor *C*_2_ contributes negligibly owing to its low ripple characteristics. For the boost mode, the duality holds. On the other hand, the measured CSF results are shown in Fig. 12(b), where the semiconductor device is identified as the main contributor. As the bias voltage approaches zero, switch *S*_1_ operates with a duty cycle nearing 100%, causing a sharp drop in the total CSF from 1 to 0.

### Evaluation of the PSFB-based SUPPC

**Fig. 13:**
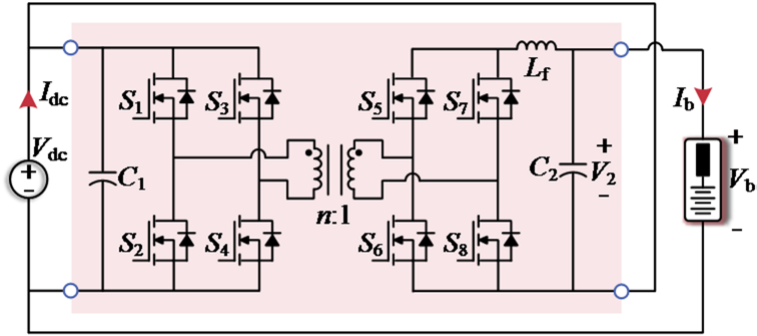
Schematic diagram of PSFB-based SUPPC.

In the study conducted by Mira et al., the PSFB converter was demonstrated to be more suitable for PPC implementation than the dual active bridge converter, primarily due to its lower cumulative CSF^31^. As shown in Fig. 13, the PSFB converter is configured using the optimal SU-A scheme, with the dc bus voltage maintained at 40 V to ensure an identical bias voltage swing. A single phase-shift control strategy is utilized to maintain a positive SP voltage, yielding a 320 W SUPPC solution. The transformer is designed with a maximum turns ratio of *n* = 2.08, under identical control margins as the SUDPPC.

**Fig. 14:**
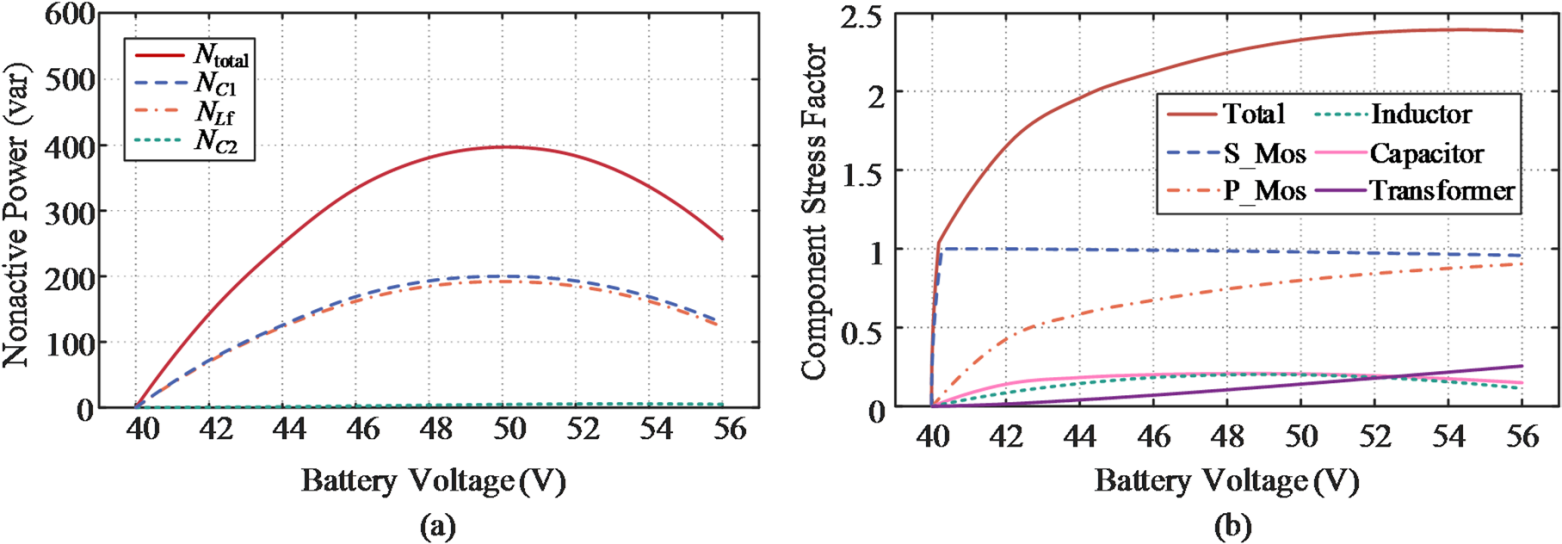
Detailed distribution of performance metrics for the PSFB-based SUPPC. (**a**) Nonactive power. (**b**) CSF.

The total nonactive power of the PSFB converter along with its component wise distribution is illustrated in Fig. 14(a). A peak nonactive power of 397 var is observed at V_b_≈50 V, which is attributed to the maximum current ripple at 0.5 phase shift ratio. Moreover, as illustrated in Fig. 14(b), the total CSF value sharply drops to zero due to the loss of the blocking voltage across the secondary-side switches during the zero-voltage transition.

### Evaluation of the SUDPPC case

**Fig. 15:**
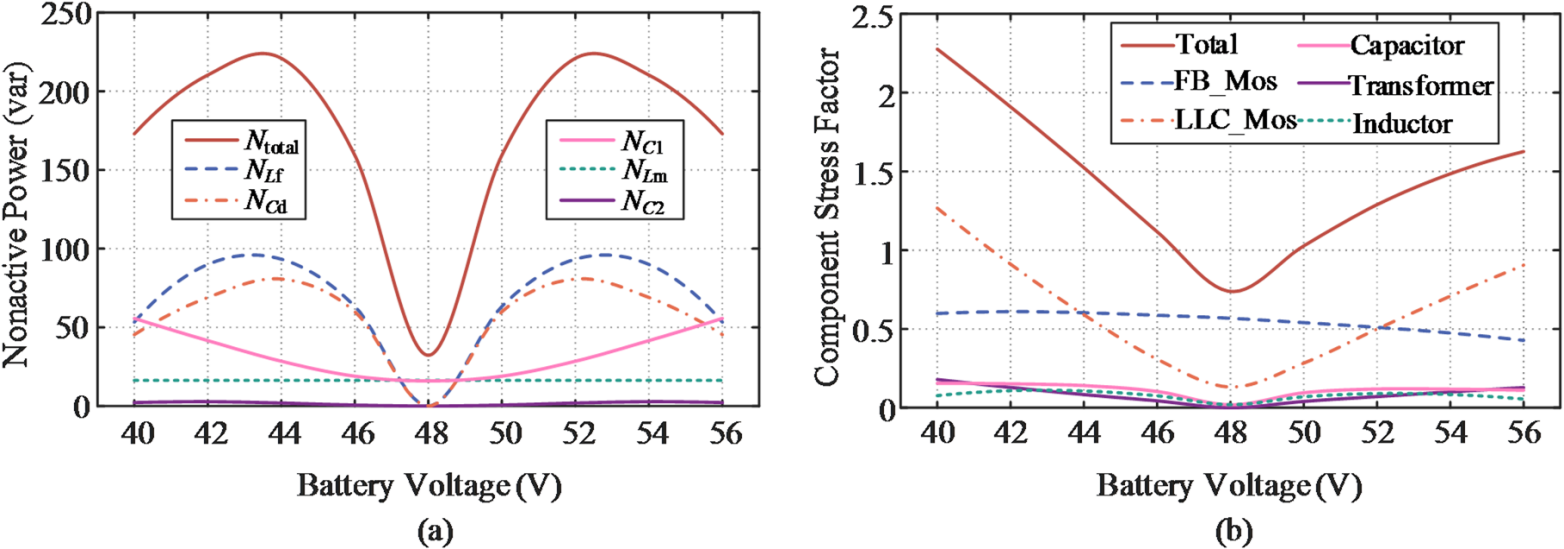
Evaluation of performance metrics for the proposed topology with *n* = 5. (**a**) Distribution of nonactive power. (**b**) Distribution of CSF.

Figure 15(a) illustrates the nonactive power characteristics for the analyzed case with a transformer turns ratio of *n* = 5. It is evident that the nonactive power is predominantly contributed by the rear-stage FB converter. In the LLC converter, the series impedance formed by the resonant capacitor *C*_r_ and inductor *L*_r_ is effectively nullified, facilitating energy transfer exclusively through the internal electromagnetic field, thereby rendering its contribution to nonactive power negligible. Under zero bias voltage, the LLC converter operates independently of the load, with total nonactive power mainly determined by the dc bus capacitor *C*_1_ and the magnetizing inductor *L*_m_. Importantly, given the soft-switching conditions achieved, the nonactive power produced by *L*_m_ remains constant across the entire voltage range. The CSF values associated with each element within the SUDPPC are revealed in Fig. 15(b), where increased component utilization is evident during step-up operation. This behavior results from the relatively lower PPF exhibited by the SUDPPC in boost mode. Owing to the capability to accommodate the full voltage range, the switches are subjected to both voltage and current stresses even during zero-voltage transition conditions.

### Comparative results analysis

**Fig. 16:**
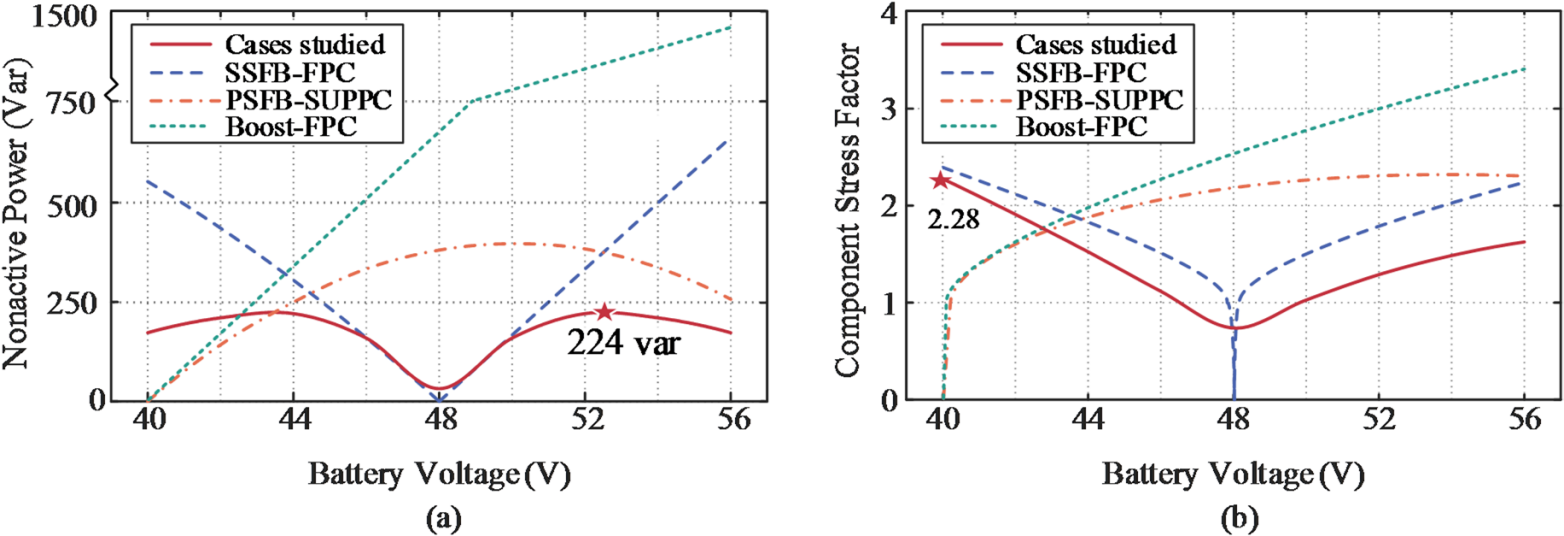
Comparison of performance metrics for different topologies. (**a**) Nonactive power. (**b**) CSF.

Figure 16 presents the comparison results of the evaluated topologies, where a standard boost converter with 40 V input is additionally tested as a baseline reference for the PSFB-based SUPPC. As clearly depicted, the properly engineered PPC significantly outperforms the FPC under identical operating conditions. Notably, the SUDPPC case presented demonstrates superior overall performance, with a maximum nonactive power of only 224 var, representing a 66.3% reduction compared to the FPC. Meanwhile, a similar behavior is observed in the CSF results, where a value as low as 2.28 under full-load conditions indicates markedly reduced component stress, thereby minimizing long-term degradation and enhancing system reliability.

**Fig. 17:**
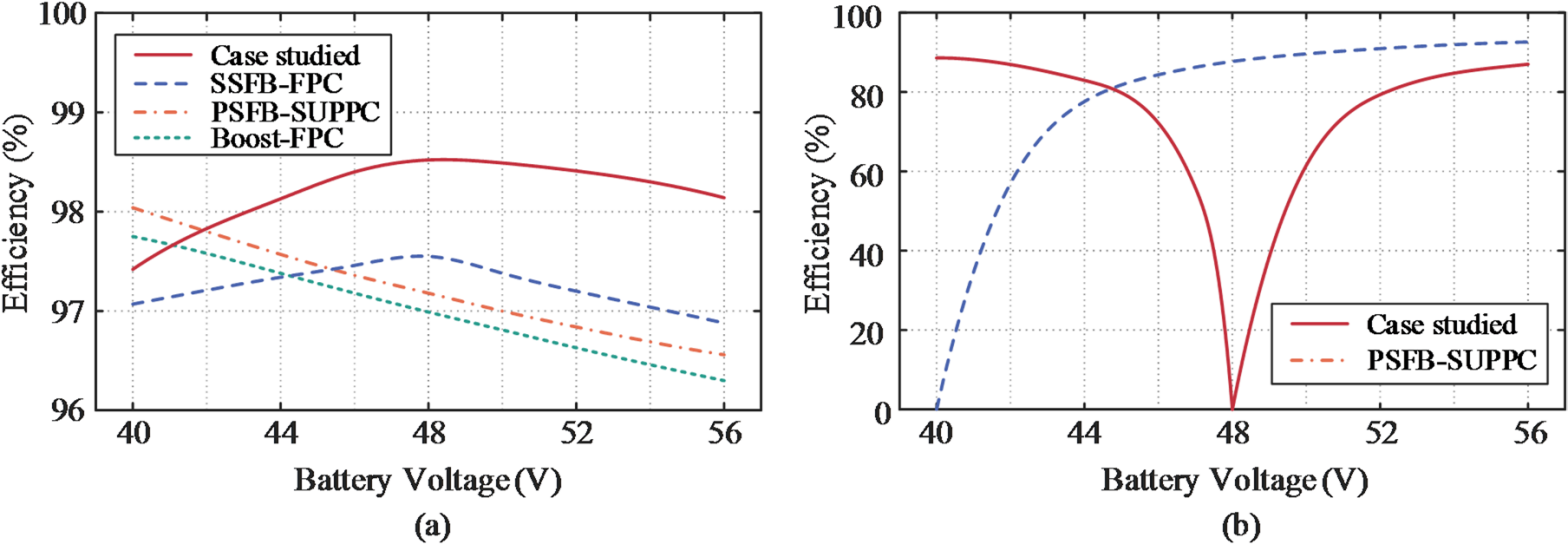
Measured terminal efficiency curves. (**a**) Overall efficiency of the dc-dc system, where the average efficiency of the four topologies from top to bottom is 98.63%, 97.4%, 96.82% and 96.64% respectively. (**b**) PPC efficiency.

Based on the operating characteristics of the evaluated topologies, the switching device stresses are summarized in Tables 3, 4 and 5, and corresponding prototype components were selected with appropriate design safety margins. Notably, the SUDPPC substantially reduces current stress at the parallel port and voltage stress on the SP terminal. By integrating detailed thermal models of components into the SIMPLIS simulation environment, terminal efficiency curves across the full output voltage range are illustrated in Fig. 17, capturing both converter-level and system-level efficiencies. The reported values are deemed reasonable for the discussed topologies. Under identical operating conditions, systems designed using the PPP methodology consistently exhibit higher overall efficiency, despite the configured PPC showing lower.

**Fig. 18:**
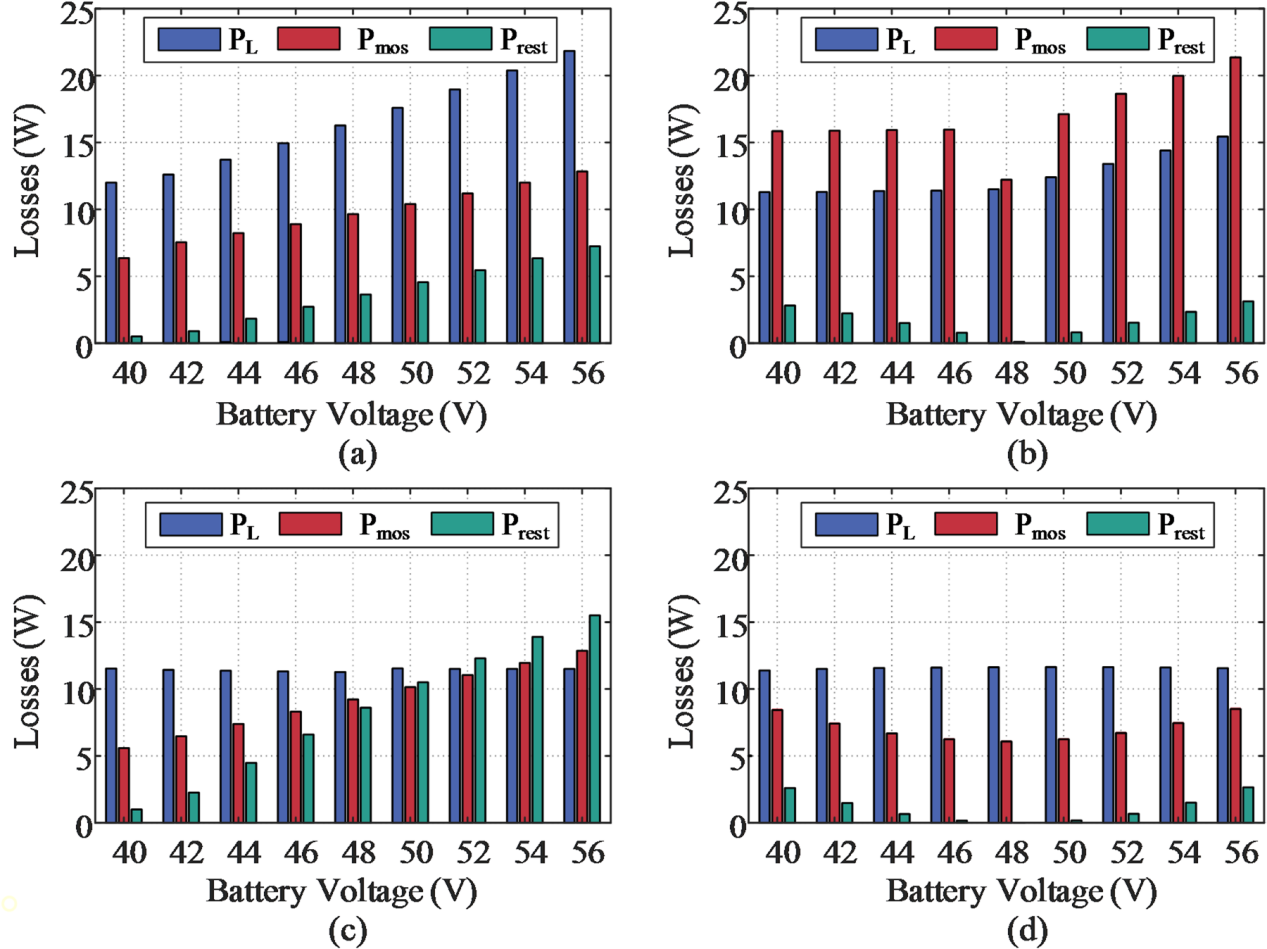
Power losses breakdown during battery charging for different topologies. (**a**) Boost converter. (**b**) FSBB converter. (**c**) PSFB-based SUPPC. (**d**) Proposed SUDPPC.

**Table 7:** Loss calculation model of main components.

Calculation model	Parameter
${P_{{\mathrm{con}}}}=I_{{{\mathrm{ds}}rms}}^{2}{R_{{\mathrm{ds}}}}$	*R*_ds_: on-resistance
${P_{{\mathrm{sw}}}}=0.5{V_{{\mathrm{ds}}}}{I_{{\mathrm{ds}}}}{f_s}({t_{{\mathrm{on}}}}+{t_{{\mathrm{off}}}})$	*t*_on_/*t*_off_: switching times
${P_{\operatorname{coss} }}=0.5V_{{{\mathrm{ds}}}}^{{\mathrm{2}}}{C_{{\mathrm{oss}}}}{f_s}$	C_oss_: output capacitor
${P_{{\mathrm{drive}}}}={V_{gs}}{Q_{gs}}{f_s}$	*V*_gs/_*Q*_gs_: gate voltage/charge
${P_{L{\mathrm{f}}}}=I_{{L{\mathrm{frms}}}}^{2}{R_{L{\mathrm{f}}}}$	*R*_*L*f_: ESR
${P_{{\mathrm{rest}}}}=I_{{{\mathrm{Crms}}}}^{2}{R_{\mathrm{C}}}+I_{{{\mathrm{Trms}}}}^{2}{R_{\mathrm{T}}}$	*R*_C_/*R*_T_: ESR

terminal efficiencies at extreme operating points. These low-efficiency points are attributed to extreme voltage conversion ratios, where the converters typically operate with low PPF values, consequently posing minimal impact on the global system efficiency. As demonstrated by Fang et al.^35^, the battery charging efficiency across the full SOC range can be converted into a time-weighted average, with the corresponding results indicated in the figure captions. Despite incorporating additional components, the SUDPPC delivers superior performance compared to both the FPC and SUPPC during the battery charging process, attaining an average efficiency of 98.63%.

Finally, the loss breakdown of the evaluated topologies is visualized in Fig. 18, encompassing the switching device loss (*P*_mos_), inductor loss (*P*_L_), and residual loss (*P*_rest_). Here, *P*_rest_​ includes the losses associated with the capacitors and the transformer. *P*_mos_ can be further divided into conduction loss *P*_con_, switching loss *P*_sw_​, output-capacitance loss *P*_coss_​, and gate-drive loss *P*_drive_​​. The corresponding calculation models are summarized in Table 7, where the RMS currents required for evaluating the losses of key components have already been provided in Sect. 3. It is evident that the dominant loss in the SUDPPC is the copper loss of the inductor, as the current flowing through the filter inductor on the series side is approximately the load current. Meanwhile, the reduced voltage stress enables the use of power devices with lower on-resistance. On the parallel side, the current is reduced by the transformer turns ratio. Compared with the conventional FPC, the PPC improves overall efficiency primarily through a significant reduction in *P*_mos_​.

### Literature review

The design of a PPC revolves around two aspects, namely the connection configuration and the internal dc-dc converter topology. To clearly illustrate the advantages and limitations of the proposed SUDPPC, a comparison with previously reported PPCs is provided in Table 8 in terms of voltage gain, number of power devices, device stress, and control complexity. It can be observed that PPCs are predominantly applied in energy systems where inherent voltage variations exist, and the achievable power level and voltage-stress reduction depend on specific operating conditions. Among existing studies, the SU-A configuration implemented in unidirectional step-up dc-dc converter systems is the most widely adopted. The topologies of PPCs are mainly categorized as dual-active-bridge (DAB) and PSFB structures. In contrast, resonant-converter-based PPCs and PPCs for multi-port systems remain scarcely investigated and represent promising future research directions. It is worth noting that the use of bipolar outputs can further reduce the rated converter power and is gradually becoming mainstream. Although the SUDPPC requires a relatively higher number of power devices, favorable key performance indicators and high overall efficiency can be achieved through appropriate parameter design.

**Table 8:** Comparison of PPC series operation.

Type	Topology	Power level (W)	Voltage gain	SP voltage stress	Power devices	Control design	Application
SU-A^6^	DAB	720	[1, 3]	0.67 *V*_o_	8	Simple	Energy Storage
SU-A^10^	PSFB	750	[0.87, 1.18]	0.15 *V*_o_	8	Complex​	PV
SU-A^14^	PSFB	750	[0.87, 1.18]	0.15 *V*_o_	8	Complex​	PV
SU-A^15^	PSFB	950	[0.85, 1.15]	0.18 *V*_o_	8	Complex	PV
SU-A^18^	DAB	5 M	[-0.55, 0.6]	0.27 *V*_o_	12	Medium	Wind farm
SU-A^20^	LLC	300	[1.85, 3.43]	0.7 *V*_o_	8	Medium	PV
SU-A^21^	Boost	3500	[1, 3]	0.67 *V*_o_	4	Simple	PV
SU-A^22^	DAB	5700	[0.84, 1.19]	0.19	10	Complex	Energy storage
SU-A^36^	PSFB	22,000	[0.86, 1.22]	0.18 *V*_o_	8	Complex	Battery charger
SU-A^37^	PSFB	1000	[0.92, 1.1]	0.09 *V*_o_	10	Complex	Energy storage
SU-A^38^	LLC	300	[1, 1.33]	0.25 *V*_o_	6	Simple	PV
SU-B^33^	Flyback	820	[1.05, 2.5]	2.3* V*_in_	3	Simple	PV
SD-A^31^	PSFB	3500	[0.6, 0.96]	0.67 *V*_o_	8	Simple	Energy Storage
SD-A^34^	SEPIC	0.1 M	[0.6, 0.96]	0.67 *V*_o_	2	Simple	Battery charger
SD-B^19^	PSFB	2000	[0.91, 1.09]	0.09 *V*_in_	10	Complex	PV
SD-B^39^	PSFB	2000	[0.91, 1.09]	0.09 *V*_in_	10	Complex	PV
SD-B^29^	Reconfigurable	2200	[0.67, 1.64]	0.64* V*_in_	16	Complex	Battery charger
SD-B^40^	DAB	17,000	[0.59, 0.77]	0.41* V*_in_	8	Simple	Battery charger
SD-A	Proposed	1100	[0.71, 1.17]	0.2 *V*_o_	12	Simple	Battery charger

**Fig. 19:**
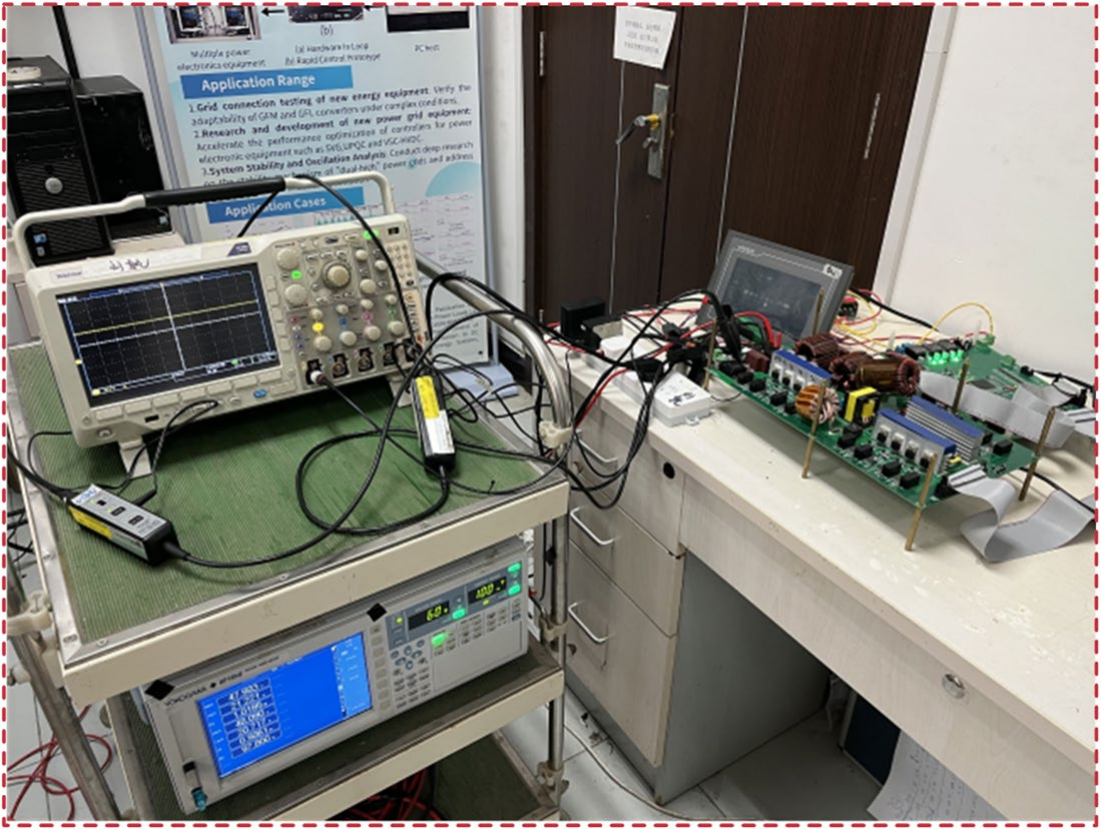
Prototype of the 1.1 kW experimental platform.

On the other hand, in the design approach presented in^14,15,36,37^, the polarity reversal of the bipolar SUDPPC leads to unavoidable oscillations in the control signals, and achieving a stable output at zero voltage remains a challenging issue. In contrast, the control logic of the SUDPPC proposed in this paper is considerably simpler, and no extreme duty cycles occur during the dynamic transition between boost and buck modes. Furthermore, zero-voltage output can be achieved by setting the duty cycle of both bridge arms to 0.5, which is obtained by sacrificing the number of power devices in the FB converter.

**Fig. 20:**
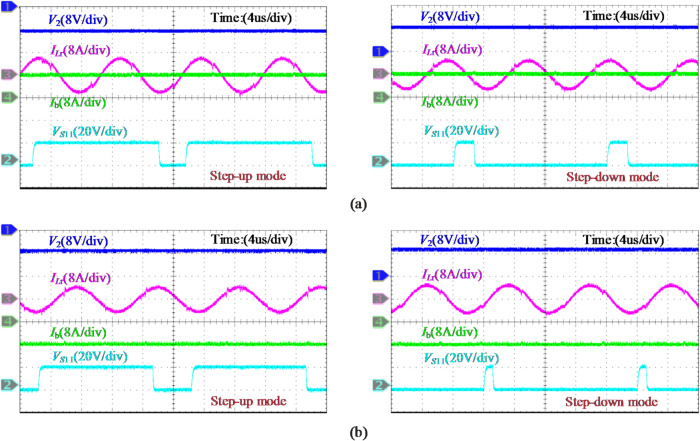
Experimental waveforms of the battery under (**a**) charging and (**b**) discharge operations, where the battery voltage *V*_b_ is set to 56 V and 40 V to describe the step-up and step-down modes, respectively.

## Experimental validation

A 1.1 kW SUDPPC prototype was built as a device between the 48 V dc bus and the battery pack, with design specifications summarized in Table 3. As illustrated in Fig. 19, the TMS320F28377 digital processor, which supports high-precision pulse-width modulation, was utilized to control the power switches. Besides, a programmable battery simulator was integrated to emulate the dynamic electrical behavior of the battery pack, allowing for controlled and repeatable testing without the need for a physical load.

Figure 20(a) displays the critical experimental waveforms of the analyzed case under the 0.4 C battery charging, which contains the SP voltage *V*_2_, resonant current *I*_*L*r_, battery current *I*_b_, and the gate signal *V*_*S*11_ for switch *S*_11_ in the FB converter. During step-up operation, characterized by a negative SP voltage, power is transferred from the dc bus to the SP side, while the reverse power flow is observed in step-down case. The duty cycle of *V*_*S*11_ for both operations is approximately 0.9 and 0.1. Similarly, the waveforms during battery discharge are visualized in Fig. 20(b), with voltage/current polarities and power flow directions as described in Fig. 5(b). The sinusoidal characteristics of *I*_*L*r_ are achieved by operating the LLC converter near the resonant frequency. At full load, the converter is measured to process 160 W, representing 14.3% of the total system active power.

**Fig. 21:**
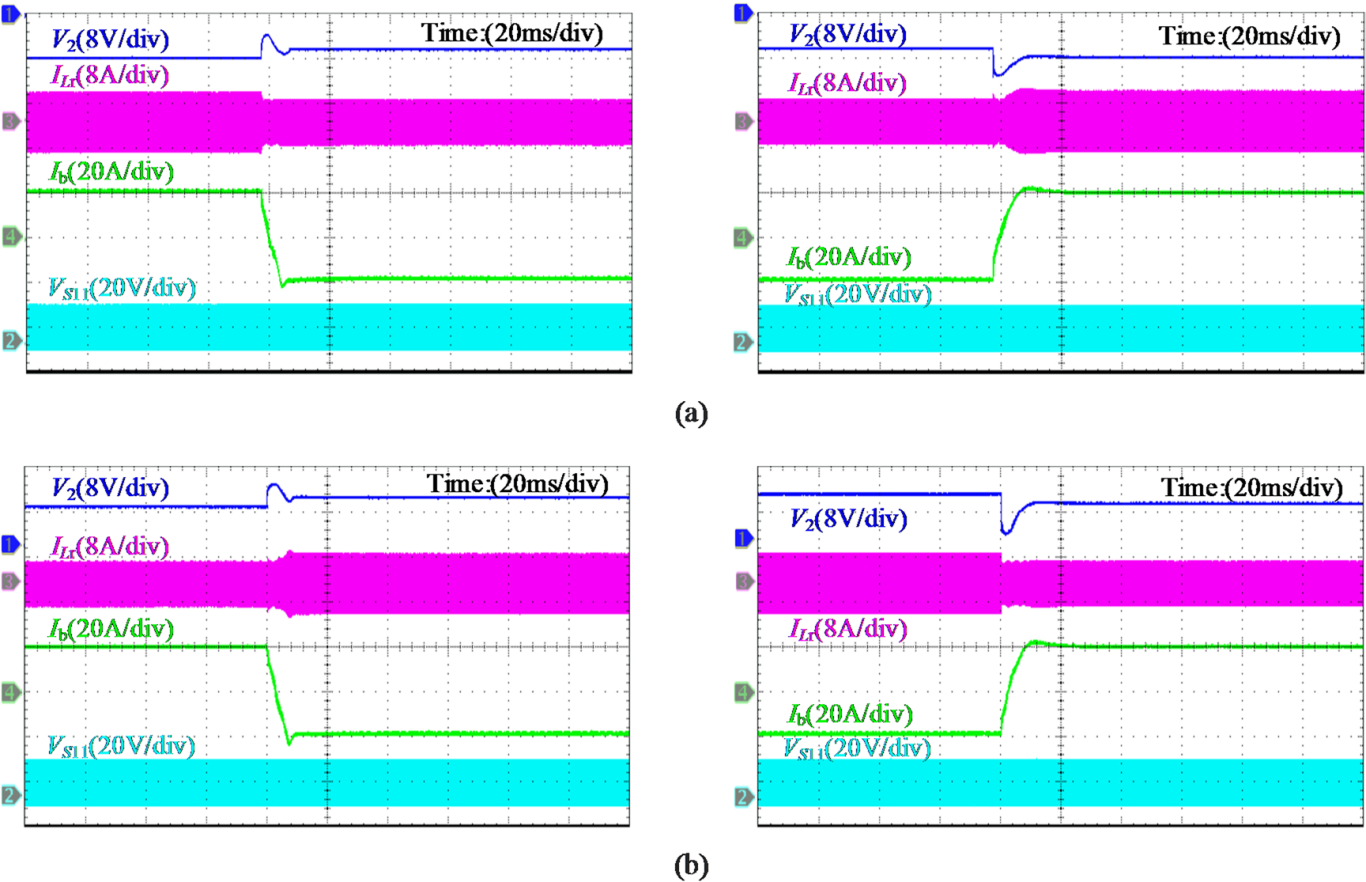
Battery current tracking performance test, where the reference value is switched between 20 A and − 20 A, with *V*_dc_=48 V. (**a**) Step-up operation *V*_b_=56 V. (**b**) Step-down operation *V*_b_=40 V.

**Fig. 22:**
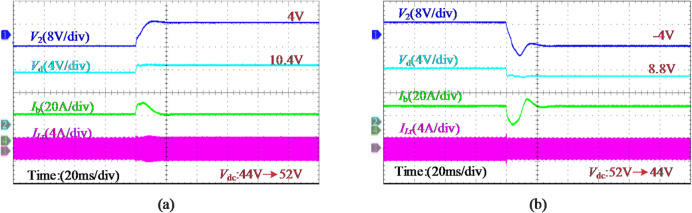
System transient response waveforms for *V*_dc_ step change, where *V*_b_ is fixed at 48 V to represent step-up/down conditions. (**a**) *V*_dc_ step-up from 44 V to 52 V. (**b**) *V*_dc_ step-down from 52 V to 44 V.

The experimental results under different reference commands are shown in Fig. 21. During the transition between charging and discharging, the battery current maintains good tracking performance, with a seamless transition completed in approximately 5 ms. Due to the direction of battery current and the effect of internal impedance, a slight fluctuation in the SP voltage amplitude is observed. Furthermore, the transient response induced by dc bus voltage variation is illustrated in Fig. 22, where *V*_dc_ changes stepwise between 44 V and 52 V. It is evident that the input voltage *V*_d_​ of the FB converter and the SP voltage *V*_2_​ vary simultaneously and stabilize following closed-loop regulation by the PPC.

**Fig. 23:**
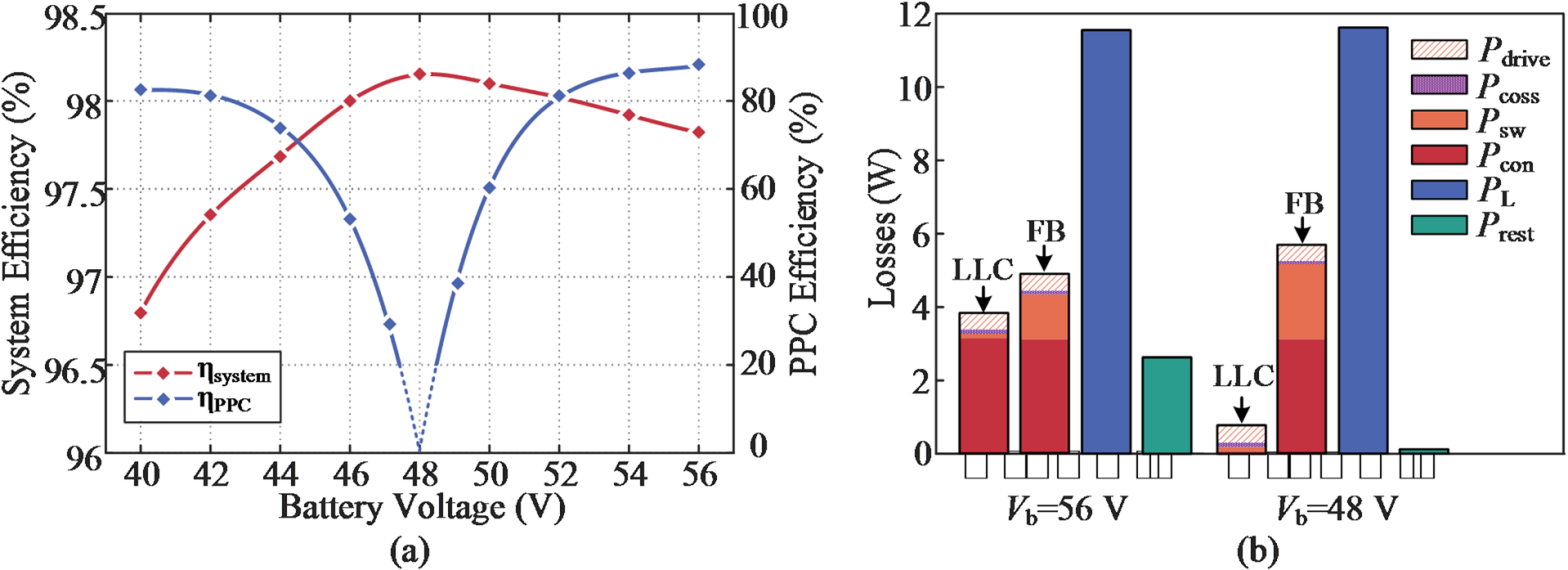
(**a**) System efficiency and PPC efficiency point captured at rated charge current. (**b**) Loss breakdown details when *V*_b_ = 48 V and 56 V.

In addition, the system efficiency and PPC efficiency when the battery is charged at 0.4 C are revealed in Fig. 23(a), which are measured using a Yokogawa WT1800 precision power analyzer. The given SUDPPC is verified to be able to maintain high efficiency over the entire SOC range, reaching a peak efficiency of 98.15% at *V*_b_=48 V. Under this condition, the SP voltage approaches zero, enabling predomi-.

-nant power transfer through the direct infeed path and filter inductor. In particular, higher global system efficiency is observed in the step-up mode due to the reduction in PPF. For battery discharge operations, essentially identical results are obtained.

Furthermore, to more clearly illustrate the loss distribution of the SUDPPC, Fig. 23(b) presents the decomposition of two representative operating points based on theoretical calculations, namely *V*_b​_=48 V and *V*_b_​=56 V. Because only minor turn-off losses are involved, the *P*_sw_ of the LLC converter is much smaller than that of the subsequent FB converter. At *V*_b​_=48 V, the battery current is transferred solely through the FB converter. During one half-cycle, switches *S*_9_ and *S*_11_​ conduct, while during the other half-cycle, *S*_10_ and *S*_12_​ conduct. Under this operating condition, the upstream LLC converter is completely decoupled from the FB stage, and no power transfer takes place, although soft switching is still maintained. As a result, the *P*_con_ of switches *S*_1​_ through *S*_8_ are eliminated, enabling operation at the peak-efficiency point. Notably, the high-efficiency advantage of the proposed SUDPPC is primarily determined by the series connection scheme and voltage matching, which provide strong robustness against variations in load characteristics and soft-switching boundaries.

## Conclusion

This paper described universal interconnection schemes for series-connected PPCs and provided guidelines based on the power distribution characteristics. A SUDPPC topology tailored for battery energy storage systems was analyzed, demonstrating that only 14.3% of the total active power is processed under full-load conditions. Its partial power processing capability was validated through derivation of nonactive power and component stress factor across the full SOC range. Comparative analysis with conventional FPC and series-connected SUPPC topologies revealed superior performance in terms of power loss and component utilization. Experimental validation using a 1.1 kW hardware prototype confirmed the theoretical analysis, with a peak efficiency of 98.15% achieved during bidirectional operation. These results indicate that PPC offers a compact and efficient dc-dc interface solution, particularly well-suited for step-up/down applications.

## Data Availability

Research data is shared if requested to the corresponding author.

## Variables

*V*_dc_: Dc bus voltage
*V*_b_: Battery voltage
*V*_2_: SP voltage
Δ*V*: Bias voltage
*I*_dc_: Dc bus voltage
*I*_b_: Battery current
*I*_Lm_: Magnetizing current
*I*_Lf_: Filter inductor current
Δ*I*_Lf_: Inductor current ripple
*P*_p_: Processed active power
*P*_d_: Direct infeed active power
*P*_max_: PPC maximum power
*P*_out_: Output active power
*f*_*s*_: LLC converter resonant frequency
*t*_*d*_: Dead time
*f*_*c*_: FB converter switching frequency
*C*_oss_: Output capacitance of the switches
*C*_r_: Resonant capacitor
*L*_r_: Resonant inductor
*L*_m_: Magnetizing inductor
*d*: FB converter equivalent duty cycle
*n*: Transformer turns ratio
